# Excessive daytime sleepiness, metabolic syndrome, and obstructive sleep apnea: two independent large cross-sectional studies and one interventional study

**DOI:** 10.1186/s12931-019-1248-y

**Published:** 2019-12-04

**Authors:** Xinyi Li, Hengye Huang, Huajun Xu, Yue Shi, Yingjun Qian, Jianyin Zou, Hongliang Yi, Jian Guan, Shankai Yin

**Affiliations:** 1 Department of Otolaryngology Head and Neck Surgery & Center of Sleep Medicine, Shanghai Jiao Tong University Affiliated Sixth People’s Hospital, Yishan Road 600, Shanghai, 200233 China; 2 Shanghai Key Laboratory of Sleep Disordered Breathing, Yishan Road 600, Shanghai, 200233 China; 3 Otolaryngological Institute of Shanghai Jiao Tong University, Yishan Road 600, Shanghai, 200233 China; 4 School of Public Health, Shanghai Jiao Tong University School of Medicine, 225South Chongqing Road, Shanghai, 200020 China

**Keywords:** Obstructive sleep apnea, Excessive daytime sleepiness, Metabolic syndrome, Upper-airway surgery

## Abstract

**Background:**

Obstructive sleep apnea (OSA) and excessive daytime sleepiness (EDS) were considered to contribute to MetS. This study was performed to assess the association between MetS and EDS in two independent large-scale populations, and in subjects who underwent upper-airway surgery.

**Methods:**

A total of 6312 patients without self-reported depression and 3578 suspected OSA patients were consecutively recruited, during health screening examinations and from our sleep center, respectively. A total of 57 subjects with OSA who underwent upper-airway surgery were also included. Demographic, anthropometric, biochemical, and polysomnographic data were obtained.

**Results:**

In the health screening examination group, 233 (9.23%) women and 350 (10.93%) men had complaints of EDS. A total of 229 (7.04%) women and 1182 (36.88%) men met the criteria for MetS. In the OSA group, 147 (21.18%) women and 1058 (36.69%) men reported EDS. In addition, 93 (13.4%) women and 1368 (47.43%) men reported MetS. In the health screening examination group, EDS did not contribute significantly to MetS (OR = 1.125, 95% CI: 0.907–1.395; *p* = 0.283). In the OSA group, EDS significantly contributed to MetS (OR = 1.249, 95% CI: 1.063–1.468; *p* = 0.007); however, the results were not significant after adjusting for sleep variables (OR = 1.071, 95% CI: 0.905–1.268; *p* = 0.423). Upper-airway surgery did not affect cardio-metabolic variables in OSA patients with or without EDS.

**Conclusions:**

EDS was not associated with MetS in two independent large-scale cohorts. In addition, upper-airway surgery did not affect components of MetS in OSA patients with and without EDS.

## Backgrounds

Obstructive sleep apnea (OSA) is one of the most common sleep-disordered breathing (SDB) patterns, and is characterized by apnea, hypopnea, oxygen desaturation, and micro-arousals due to recurrent episodes of partial or complete upper airway collapse during sleep [1]. The ensuing cardinal symptoms of OSA, such as excessive daytime sleepiness (EDS) can persist throughout the day, leading to depression [2], low productivity and poor work performance [3], increased risk of traffic accidents and related deaths [4], and impaired quality of life [5].

EDS is also a common problem, with a prevalence of 9.4% in the Chinese population according to an Asian multi-ethnic study [6]. The prevalence of EDS in patients with OSA is even higher, at 16–22% [7]. The high prevalence of EDS imposes a social and economic burden worldwide. Recently, several clinical studies have shown that EDS is associated with individual components of metabolic syndrome (MetS) such as obesity [8], insulin resistance, [9, 10] and hypertension [11]. However, few studies have explored the relationship between EDS and MetS as a complete entity, rather than in terms of its individual components. One community study [12] and two clinical studies [13, 14] assessed the relationship between EDS and MetS; however, the findings should be interpreted with caution because the definition of MetS in the community study focusing on the general population was inaccurate, where fasting serum glucose, triglycerides (TG), and high-density lipoprotein-cholesterol (HDL-c) were not measured [12]. The two cross-sectional studies focused on males and patients with severe OSA, respectively, and the results may therefore have been biased [13, 14]. The relationship between MetS and EDS in patients with or without OSA is complex and poorly understood.

Continuous positive airway pressure (CPAP) is the first-line treatment for OSA and can decrease levels of cholesterol, insulin, and the insulin resistance index in subjects with EDS, although it does not affect these variables in subjects without EDS [15]. Upper-airway surgery is another common therapeutic method for OSA that can improve daytime sleepiness in nonobese patients with OSA, especially in patients with severe OSA [16]. In a retrospective case review, upper-airway surgery improved blood lipid levels in patients with OSA [17]. However, whether abolishment of nocturnal apneas by upper-airway surgery improves metabolic status in patients with or without EDS remains unclear.

In this study, we explored whether EDS is independently associated with MetS in three independent groups: 1) a general population (subjects undergoing health screening examinations), 2) a suspected OSA population, and 3) OSA patients that underwent upper-airway surgery.

## Methods

### Participants

#### Cohort 1

Between March 2011 and September 2011, a total of 9310 subjects without self-reported depression were consecutively recruited during health screening examinations without PSG diagnosis conducted at the Shanghai JiaoTong University Affiliated Sixth People’s Hospital. The final sample comprised 6312 participants. The exclusion criteria presented in Supplementary Material and Data 1.1.

#### Cohort 2

A total of 4730 patients suspected to have OSA were consecutively enrolled from July 2007 to July 2015 with overnight PSG (Alice 4 or 5; Philips Respironics, Pittsburgh, PA, USA) at our sleep center. A total of 3578 subjects were included in the final analysis. The exclusion criteria presented in Supplementary Material and Data 1.2.

#### Cohort 3

Between January 2013 and January 2014, patients with OSA who had undergone upper-airway surgery [uvulopalatopharyngoplasty], recruited from our sleep center, were included. All participants underwent polysomnography (PSG) monitoring before upper-airway surgery and after the 6-month follow-up. Exclusion criteria included: 1) severe comorbidities, 2) receiving other treatments for OSA after upper-airway surgery, and 3) taking anti-metabolic medications. Finally, 57 subjects were included in the statistical analysis.

Written informed consent was obtained from all subjects. Our studies were performed according to the Declaration of Helsinki and were approved by the Ethics Committee of Shanghai Jiao Tong University Affiliated Sixth People’s Hospital.

### Daytime sleepiness assessment

Daytime sleepiness was evaluated using the validated ESS questionnaire, as described previously [18]. The ESS includes eight questions quantifying the individual’s sleep propensity and likelihood of “dozing” (0 = never, 1 = slight, 2 = moderate, and 3 = high probability) in different situations. The sum of the ESS score ranges from 0 to 24, with higher scores representing a higher degree of sleepiness. A cutoff EDS score of > 10 was defined as EDS [19].

### Metabolic syndrome

Anthropometric measurements seen Supplementary materials and methods 1.3. Fasting blood samples were drawn from every participant in the morning. Weight, height, neck circumference (NC), waist circumference (WC), and hip circumference (HC) were measured following standardized procedures. Waist-hip ratio (WHR; WC/ HC), and body mass index (BMI; weight/height2) were calculated. Serum fasting glucose and serum fasting lipid profiles [i.e., total cholesterol (TC), TG, high density lipoprotein (HDL), and low density lipoprotein (LDL)] were measured using an H-7600 autoanalyzer (Hitachi, Tokyo, Japan) in the hospital laboratory, as described previously [20]. Blood pressure were measured in triplicate after at least a 5-min rest using an automated electronic device (Omron Model HEM-752 Fuzzy, Omron Company), and the average value of the three measurements was used for analysis. Adult Treatment Panel-III (ATP-III) criteria with the WC criteria for Asians was used to define MetS [21]. MetS was diagnosed in patients exhibiting at least three of the following metabolic features 1) WC ≥ 90 cm (in men) and ≥ 80 cm (in women); 2) TG ≥ 1.70 mmol/L; 3) HDL < 1.03 mmol/L (in men) and < 1.30 mmol/L (in women); 4) systolic blood pressure (SBP) ≥ 130 mmHg or diastolic blood pressure (DBP) ≥ 85 mmHg, or use of antihypertensive drugs; and 5) fasting glucose: ≥ 5.6 mmol/L or use of antidiabetic drugs. The metabolic score was given by the total number of diagnostic criteria for MetS fulfilled. Homeostasis model assessment-estimated insulin resistance (HOMA-IR) was given by (Glucose*Insulin) / 22.5. Insulin resistance was defined as a HOMA-IR index > 2.5 [22].

### Polysomnographic evaluation

In Cohorts 2 and 3, overnight PSG (Alice 4 or 5; Philips Respironics, Pittsburgh, PA, USA) was performed to assess objective sleep status according to the American Academy of Sleep Medicine (AASM) 2007 criteria [23]. For patients with OSA who underwent upper-airway surgery, preoperative PSG was performed within the 2 weeks prior to upper-airway surgery and postoperative PSG was performed at least 6 months after upper-airway surgery. The full-night PSG sessions included electroencephalogram (EEG), bilateral electrooculogram (EOG), electrocardiogram (ECG), genioglossus electromyogram, thoracic-abdominal movement, nose and mouth airflow, finger pulse oxygen saturation, bilateral leg movement, and body position measurements. Objective sleep parameters such as apnea hypopnea index (AHI), oxygen desaturation index (ODI), mean oxygen saturation (MSaO_2_), lowest pulse oxygen saturation (LSpO_2_), and micro-arousal index (MAI) were calculated using software and manually checked by a skilled technician.

Apnea was defined as a complete cessation of airflow for ≥10 s; hypopnea was defined as a ≥ 50% reduction in airflow for ≥10 s, with ≥ 3% oxygen desaturation or arousal. Arousal was taken as abrupt shifts in electroencephalographic frequency lasting at least 3 s. AHI was calculated as the mean number of apnea events and hypopnea episodes per hour during sleep. The ODI was calculated as the mean number of oxyhemoglobin desaturation ≥3% events per hour of sleep. The MAI was calculated as the mean number of arousal episodes per hour of sleep. The severity of OSA was categorized as normal (< 5 events per hour), mild (5–14.9 events per hour), moderate (15–29.9 events per hour), or severe (≥ 30 events per hour) according to the AHI.

### Statistical analysis

All statistical analyses were performed using SPSS (ver. 22.0; SPSS Inc., Chicago, IL, USA) and Analysis of Moment Structures (AMOS) software (ver. 21.0; IBM Corp., Armonk, NY, USA). All raw data were examined for normality before any statistical analysis was performed. If the distribution of any variable was skewed, it was natural logarithm-transformed. To explore the magnitude of each component and subcomponents of MetS in non-EDS and EDS patients in the OSA group, confirmatory factor analysis (CFA) was performed. The models were estimated using the maximum likelihood method. First, congruency between the hypothesized models and empirical data from the EDS and non-EDS subgroups was examined using the chi-square difference test. The fit of the models was then assessed by two incremental model fit indices [comparative fit index (CFI) and root mean-square error of approximation (RMSEA)], as the chi-square test is sensitive to sample size. If the *p*-value for chi-square > 0.05, the CFI value was > 0.9 and the RMSEA value was < 0.05 (good) or < 0.08 (rational), the CFA model was considered to have a good fit. The effects of upper-airway surgery on components of MetS were analyzed using paired t tests. A *p* value < 0.05 was considered to indicate statistical significance.

## Results

### Binary logistic regression analysis

Basic characteristics of the three cohorts with EDS and MetS were presented in Supplementary Material and Data 2.1(Additional file 1: Tables S1–S7). To assess independent factors associated with MetS in the subjects who underwent health screening examinations, the following variables were entered into the logistic regression models: EDS, age, sex, BMI, smoke and drink. MetS in subjects undergoing health screening examinations was significantly associated with the following variables: age (OR = 1.019, 95% CI: 1.014–1.024; *p* < 0.001), sex (OR = 0.189, 95% CI: 0.158–0.226; *p* < 0.001), BMI (OR = 1.235, 95% CI: 1.207–1.263; *p* < 0.001), smoke (OR = 1.432, 95% CI: 1.218–1.683; *p* < 0.001). There was no significant association with EDS (OR = 1.092, 95% CI: 0.878–1.357; *p* = 0.429 and alcohol (OR = 1.089, 95% CI: 0.928–1.279; *p* = 0.297). To increase our understanding of sex-related differences, the analysis was conducted separately in males and females. No significant association between MetS and EDS was found (OR = 1.169, 95% CI: 0.920–1.484; *p* = 0.201 in males and OR = 0.713, 95% CI: 0.397–1.281; *p* = 0.258 in females) after full adjustment.

To assess independent factors associated with MetS in patients with suspected OSA, EDS, age, sex, BMI, smoke and drink were entered into logistic regression models. The basic characteristics subjects with suspected OSA are listed in Additional file 1: Table S1. Most of subjects were OSA patients, subjects with OSA were more obese and had higher levels of glucose, lipid profiles than those without OSA (Additional file 1: Table S1). MetS in OSA was significantly associated with the following variables: sex (OR = 0.214, 95% CI: 0.163–0.282; *p* < 0.001), BMI (OR = 1.320, 95% CI: 1.287–1.355; *p* < 0.001), age (OR = 1.015, 95% CI: 1.008–1.023; p < 0.001), EDS (OR = 1.241, 95% CI: 1.055–1.460; *p* = 0.009) and smoke (OR = 1.350, 95% CI: 1.146–1.592; *p* < 0.001. However, when we included sleep variables (AHI, ODI, and MAI) in the model, the association with EDS disappeared (OR = 1.068, 95% CI: 0.902–1.264; *p* = 0.447). To explore sex differences, we also performed logistic analysis separately in males and females adjusted for age, BMI, smoke and drink. In males, no significant relationship between MetS and EDS was found (OR = 1.110, 95% CI: 0.930–1.326; *p* = 0.247) after full adjustment, similar to females (OR = 0.665, 95% CI: 0.361–1.225; *p* = 0.189).

### Confirmatory factor analysis (CFA)

#### Model estimation and evaluation of the whole OSA group (model 1, Fig. 1)

**Fig. 1:**
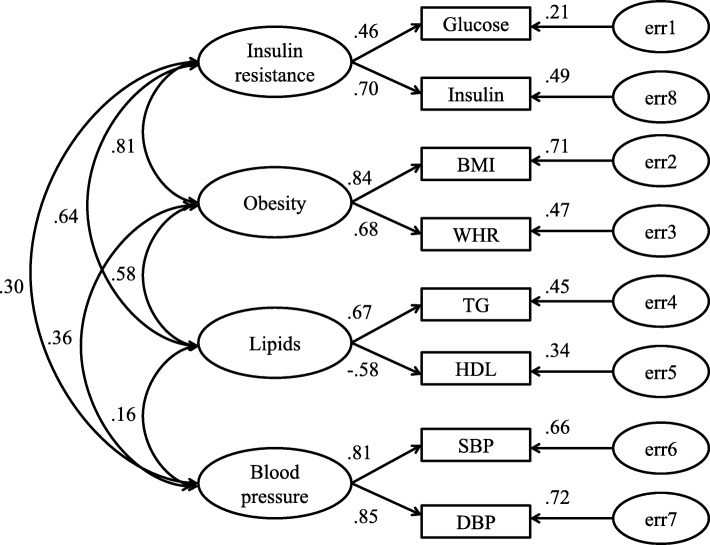
Model estimation of metabolic risk factors associated with OSA (model 1). Hierarchical 4-factor model of MetS for OSA. CMIN = 299.032, df = 21, *n* = 3578, *p* < 0.001, NC = 14.240, CFI = 0.972, RMSEA = 0.061. ‘e’ represents residual covariance

**Fig. 2:**
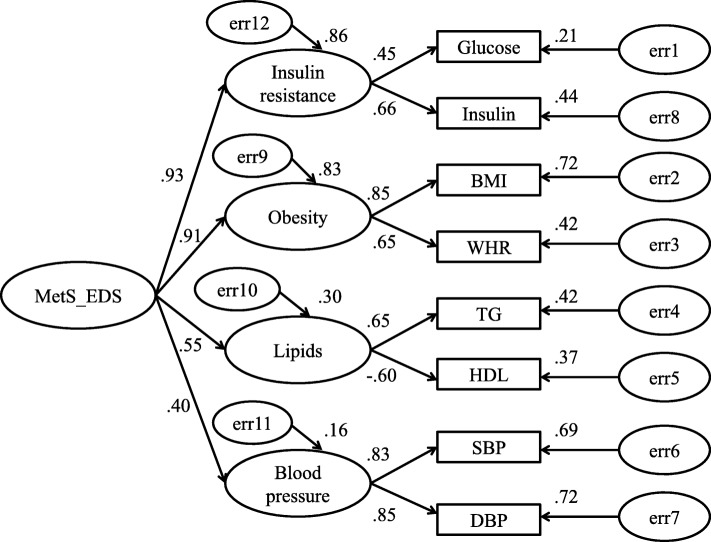
Model estimation of metabolic risk factors associated with EDS (model 2). Correlated 4-factor model for the EDS. CMIN = 133.67, df = 23, *n* = 1205, *p* < 0.001, NC = 5.812, CFI = 0.966, RMSEA = 0.063). ‘e’ represents residual covariance

**Fig. 3:**
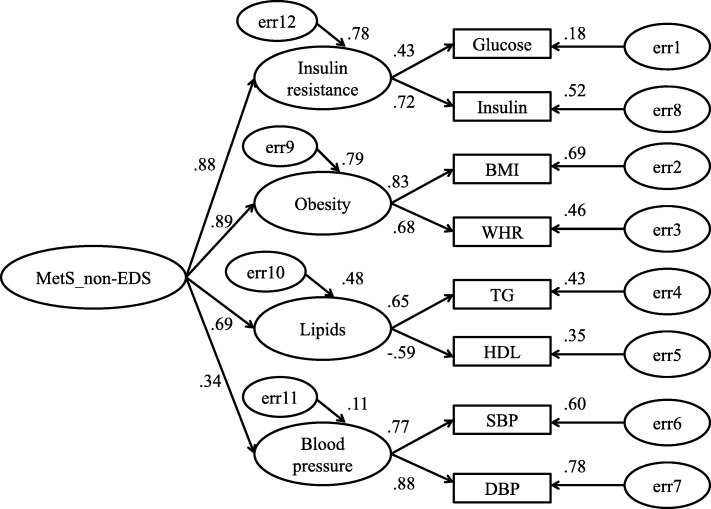
Model estimation of metabolic risk factors associated with non-EDS (model 3). Correlated 4-factor model for the non-EDS. CMIN = 221.854, df = 23, *n* = 2373, *p* < 0.001, NC = 9.646, CFI = 0.969, RMSEA = 0.060. ‘e’ represents residual covariance

Measurement models of CFA are listed in Figs. 1, 2 and 3. In these figures, insulin resistance, obesity, lipids and blood pressure are latent independent variables, and glucose, insulin, BMI, WHR, TG, HDL, SBP, DBP were observed indicators. Straight arrows represented causation relationship and double-headed curved arrows are correlations between indicators.

In Fig. 1, the goodness of fit results of a hierarchical four-factor model for MetS conducted in the whole OSA group are presented (CMIN = 299.032, df = 21, *n* = 3578, *p* < 0.001, NC = 14.240, CFI = 0.972, RMSEA = 0.061). Insulin resistance and obesity were strongly correlated (loading = 0.81, *p* < 0.001). The other latent independent variables were not correlated (*P* > 0.05). Insulin resistance was mainly represented by insulin (loading = 0.70, *p* < 0.001). Obesity was mainly represented by BMI (loading = 0.84, *p* < 0.001). Lipid profile was mainly represented by TG (loading = 0.67, *p* < 0.001). Finally, blood pressure was represented equally by SBP (loading = 0.81, *p* < 0.001) and DBP (loading = 0.85, *p* < 0.001).

#### Model for OSA in the EDS group (model 2, Fig. 2) and non-EDS group (model 3, Fig. 3)

Hierarchical four-order factor MetS models of CFA were created for OSA with and without EDS in Fig. 2 and Fig. 3 [(CMIN = 133.67, df = 23, *n* = 1205, *p* < 0.001, NC = 5.812, CFI = 0.966, RMSEA = 0.063); CMIN = 221.854, df = 23, *n* = 2373, *p* < 0.001, NC = 9.646, CFI = 0.969, RMSEA = 0.060, respectively]. In both the OSA with and without EDS groups, MetS was mainly represented by insulin resistance [model 2: loading = 0.93, *p* < 0.001; model 3: loading = 0.88, *p* < 0.001] and obesity [model 2: loading = 0.91, *p* < 0.001; model 3: loading = 0.89, *p* < 0.001]. Lipids and blood pressure were poorly represented in the MetS models. Furthermore, insulin resistance was mainly defined by insulin [model 2: loading = 0.66, *p* < 0.001; model 3: loading = 0.72, *p* < 0.001], and obesity by BMI [model 2: loading = 0.85, *p* < 0.001; model 3: loading = 0.83, *p* < 0.001].

To assess the independent relationships of HOMA-IR and obesity with EDS in the OSA group, the following variables were adjusted for: age, sex, and sleep variables (AHI, ODI, and MAI). EDS significantly predicted obesity (OR = 1.230, 95% CI: 1.034–1.463; *p* = 0.020), but not HOMA-IR (OR = 1.026, 95% CI: 0.875–1.204; *p* = 0.750).

#### Effects of upper-airway surgery on components of MetS in patients with OSA, with and without EDS

Table 1 presents the effects of upper-airway surgery on components of MetS. In OSA subjects with EDS, there was a small but non-significant change in almost all variables. In contrast, in OSA without EDS, upper-airway surgery did not affect the variables. Thus, there was no differential effect of upper-airway surgery in OSA with and without EDS (all *p* > 0.05).

**Table 1:** Effects of upper-airway surgery on components of MetS in patients with *OSA* with and without *EDS*

	OSA without EDS (*n* = 24)		OSA with EDS (*n* = 33)		Differences in after-before upper-airway surgery between patients with and without EDS (95%CI)	*p* value
	Before upper-airway surgery	After upper-airway surgery	Before upper-airway surgery	After upper-airway surgery		
Glucose(mmol/L)	5.43(0.72)	5.00(1.48)	5.28(0.69)	5.03(1.14)	−0.28(− 0.76~0.21)	0.61
Insulin(μU/mL)	10.50(4.95)	10.65(4.83)	15.83(11.05)	15.66(8.00)	−1.81(−4.51~0.89)	0.94
HOMA-IR	3.39(2.59)	2.79(2.14)	3.93(2.56)	3.33(2.86)	−0.60 (−1.21~0.01)	0.70
TC(mmol/L)	4.93(0.76)	4.83(0.84)	4.96(1.02)	4.57(0.76)	−0.33(−0.54~ − 0.12)	0.69
TG(mmol/L)	2.16(1.98)	2.55(3.69)	2.74(2.42)	2.19(0.95)	−0.03(− 0.82~0.76)	0.91
HDL(mmol/L)	1.06(0.21)	1.00(0.19)	1.14(0.29)	1.09(0.24)	−0.04(− 0.09~0.01)	0.70
LDL(mmol/L)	3.29(0.83)	2.95(0.65)	3.14(0.51)	2.87(0.63)	−0.31(− 0.51~ − 0.11)	0.86
SBP(mmHg)	126.00(13.23)	128.86(14.78)	128.80(15.88)	123.90(14.45)	−2.41(−8.75~3.93)	0.34
DBP(mmHg)	81.21(7.10)	82.64(11.02)	81.60(10.28)	78.15(9.09)	0.88(−5.23~7.03)	0.56
BMI(kg/m2)	28.26(4.93)	27.68(3.82)	28.36(2.85)	27.82(3.35)	−0.67(−1.05~ − 0.30)	0.38
WHR	0.96(0.01)	0.95(0.01)	0.97(0.06)	0.97(0.05)	−0.01(−0.03~0.01)	0.67

Abbreviations: *OSA*, obstructive sleep apnea; *EDS*, excessive daytime sleepiness; *BMI*, body mass index; *HOMA-IR*, homeostasis model of assessment for insulin resistance index; *TC*, total cholesterol; *TG*, triglycerides; *HDL-C*, high-density lipoprotein cholesterol; *LDL-C*, low-density lipoprotein cholesterol; *SBP*, systolic blood pressure; *DBP*, diastolic blood pressure

## Discussion

In two independent large-scale cohorts, MetS did not differ according to the presence or absence of EDS in this study. The results showed that upper-airway surgery did not modify components of MetS in OSA with and without EDS. These findings suggest that EDS may not be a useful clinical marker for MetS.

In this study, the prevalence of EDS differed between the health screening examination group and OSA group (9.2 and 33.7%, respectively). The prevalence of EDS in the general population in our study was similar to previous reports [6] in Asian subjects. However, the prevalence of EDS was higher in our study than in previous studies, as most subjects experienced severe OSA [7]. The fact that our severe OSA patients suffered more with oxygen desaturation and fragmented sleep (micro-arousals) may explain the higher percentage of EDS in our study.

With regard to the relationship between EDS and MetS, findings have been inconsistent. In one study, EDS was significantly associated with both OSA and MetS [24]. In another investigation, daytime sleepiness in obese subjects was associated with metabolic abnormalities [8]. Also, Bixler et al. showed that EDS was more strongly associated with metabolic factors than with OSA [25]. However, other studies have shown different results. One study found that daytime sleepiness was associated with nocturnal sleep disturbance, but not OSA and markers of MetS, in severely obese subjects [26]. However, elsewhere, EDS did not affect the relationship between OSA and metabolic variables [10]. Symptoms of EDS were not associated with sympathetic nervous system activation or arterial stiffness in non-severe OSA [27]. Furthermore, no relationship was found between EDS and stroke risk, and only weak associations were found between EDS and risk of cardiovascular and coronary heart disease [28]. MetS is an independent risk factor for pre-cardiovascular disease (CVD) and CVD; EDS may not be associated with MetS, a view consistent with our findings.

In both our EDS and non-EDS OSA subjects, obesity and insulin resistance were the main factors associated with MetS in CFA. In further analysis, EDS was independently associated with obesity. An independent relationship between EDS and obesity was also been found in previous studies [29, 30]. One study found that EDS was associated with greater risk of central obesity, independent of diet and physical activity [29]. Another study showed that weight gain had a detrimental effect on daytime sleepiness, mostly through pathways other than OSA [30]. Weight loss interventions improve daytime sleepiness, supporting the hypothesized causal effect of obesity on daytime sleepiness [31]. Furthermore, we found an independent relationship between these variables even after adjusting for objective sleep parameters. The presence of EDS is associated with depressive symptoms, but not with SDB [32]. No significant difference in EDS between SDB and non-SDB subjects was found [33]. The lack of an association between EDS and OSA may explain why OSA did not affect the relationship between EDS and obesity.

### Limitations

Strengths of our study included the inclusion of two large independent cohorts, and one retrospective interventional cohort, which allowed us to clearly address the relationship between EDS and MetS. Nevertheless, several limitations of the current study should be addressed. First, depression is closely associated with EDS; although we excluded subjects shown to be depressed according to self-report measures, physician diagnosis, or anti-depression treatment status, we did not perform a clinical interview to diagnose depression. Second, we used a subjective questionnaire, the ESS, to assess EDS rather than an objective method, such as the multiple sleep latency test (MSLT). However, whether MSLT represents the gold standard to diagnose EDS remains unclear [34]. Third, visceral adipose tissue is an important factor in the development of MetS and EDS, but we measured only WHR as a marker of abdominal adiposity and not subcutaneous or abdominal fat. Fourth, certain other potential confounding factors, such as economic, nutritional and physical activity status, were not evaluated. Lastly, causation cannot be directly inferred when using a temporal cross-sectional design and selection bias may exist in retrospective cohort studies; thus, further prospective and randomized controlled studies are required to delineate the relationship between EDS and MetS.

## Conclusions

The cross-sectional surveys indicated that EDS is not independently associated with MetS, but is associated with obesity in OSA. Treatment via upper-airway surgery in patients with OSA did not improve MetS, with or without EDS. The study was applied to better understand the complex relationship between excessive daytime sleepiness and metabolic syndrome in OSA.

## Supplementary information

**Additional file 1.** Description and basic characteristics of the three cohorts.

## Data Availability

The corresponding authors will provide the accessibility of clinical data applied to support conclusions after receiving request.

## Abbreviations

BMI: Body mass index
DBP: Diastolic blood pressure;
EDS: Excessive daytime sleepiness; metabolic syndrome
HC: Hip circumference
HDL-C: High-density lipoprotein cholesterol
HOMA-IR: Homeostasis model of assessment for insulin resistance index
LDL-C: Low-density lipoprotein cholesterol
MetS; NC: Neck circumference
OSA: Obstructive sleep apnea
SBP: Systolic blood pressure
TC: total cholesterol
TG: Triglycerides
WC: Waist circumference
WHR: Waist-hip ratio
