# Supplementary material for: Excessive daytime sleepiness, metabolic syndrome, and obstructive sleep apnea: two independent large cross-sectional studies and one interventional study
Source: Respir Res. 2019 Dec 4;20:276. doi: 10.1186/s12931-019-1248-y (PMC6894285; doi:10.1186/s12931-019-1248-y)
Supplement: Supplementary file 1 — Additional file 1. Description and basic characteristics of the three cohorts. [file 12931_2019_1248_MOESM1_ESM.docx]

**Supplementary Material and Data**

**1. Materials and methods**

**1.1 the exclusion criteria of Cohort 1:** We excluded participants aged < 18 years (n = 3), and those who were receiving OSA treatment (n = 2), were suffering from systemic diseases (n = 24), had missing glucose data or were undergoing antidiabetic therapy (n = 233), had missing lipid profile data or were receiving lipid-lowering treatment (n = 2557), had missing blood pressure data or were taking anti-hypertension drugs (n = 139), had no Epworth Sleepiness Scale (ESS) data (n = 22), or had no waist circumference (WC) data (n = 18).

**1.2 the exclusion criteria of Cohort 2:** 1,152 were excluded for the following reasons: 1) aged < 18 years (n = 76), 2) presence of systematic diseases (chronic kidney disease, congestive heart failure, hepatic failure, or chronic pulmonary disease), immune diseases, cancer, psychiatric diseases, or infectious diseases (n = 81), 3) previously treated for OSA via upper-airway surgery and CPAP (n = 214), 4) had taken lipid-lowing, anti-hypertension, or diabetes medications (n = 482), and 5) had missing data (n = 267). Patients with depression or other sleep disorders, such as upper-airway resistance syndrome, narcolepsy, periodic limb movement disorder, or restless legs syndrome were also excluded (n = 32).

1.3 *Anthropometric measurements and definition*

Well-trained examiners measured weight, height, neck circumference (NC), WC, and hip circumference (HC) following standardized procedures. We used WC and HC to calculate the waist-hip ratio (WHR; WC/ HC), and used weight and height to calculate the body mass index (BMI; weight/height^2^). Obesity was defined as BMI ≥ 28 kg/m^2^ according to the World Health Organization recommended BMI cut-off point for Asian populations[[1](#_ENREF_1)]. Weight was measured using an electronic scale and height was measured using a tape measure with participants in a standing position. Both weight and height were obtained under barefoot and light clothing conditions. NC was measured at the level of the laryngeal prominence; WC was measured at the midpoint between the bottom of the rib cage and the top of the iliac crest, following exhalation; HC was measured over thin clothing at the point of maximum circumference of the buttocks.

1.4 *Statistical analysis*

All statistical analyses were performed using SPSS (ver. 22.0; SPSS Inc., Chicago, IL, USA). Data are presented as the median (interquartile range) [skewed], mean and standard deviation [normally distributed], or number (percentage) [categorical]. Normally distributed and skewed data were analyzed using the independent sample t test and Mann–Whitney U test, respectively. Categorical variables were analyzed using the chi-square test or Fisher’s exact test. Multivariate logistic regression models were performed to assess determinants of MetS and EDS. Age, anthropometric, and sleep variables were tested sequentially.

**2. Results**

***2.1Basic characteristics***

*Basic characteristics of women*

Demographic data for the women with and without EDS are presented in Tables 1S and 4S. In the health screening examination group (Table 1S), 233 (9.23%) women had complaints of EDS. The median age of the women was 53.0 years (interquartile range: 41–57 years), and no difference in age was found between those with and without EDS. No differences were detected in obesity indices (i.e., BMI, NC, WC, HC, and WHR) between women with and without EDS. Excluding DBP and TC, levels of other cardio-metabolic parameters were similar between the two groups (women with and without EDS). In the OSA group (Table 4S), 147 (21.18%) women reported EDS, with a median age of 45 years (interquartile range: 35–55 years). Women who reported EDS were slightly older, with a higher rate of obesity and worse cardio-metabolic and sleep status.

The basic characteristics of subjects with suspected OSA are shown in Table 3S. Most of subjects were OSA patients, subjects with OSA were more obese and had higher levels of glucose, lipid profiles than those without OSA. The characteristics of women with and without MetS are shown in Tables 2S and 5S. In the health screening examination group (Table 2S), 229 (7.04%) women met the criteria for MetS. Compared to those without MetS, women with MetS were older, and showed a higher rate of obesity and higher levels of cardio-metabolic variables. No difference was detected in ESS scores between the two groups. In the OSA group (Table 5S), 93 (13.4%) women had MetS. Almost all variables (excluding LDL and MAI) differed between women with and without MetS.

*Basic characteristics of men*

Demographic data for men with and without EDS are shown in Tables 1S and 4S. In the health screening examination group (Table 1S), 350 (10.93%) men complained of EDS. The median age of male participants was 49 years (interquartile range: 39–59 years). Men with EDS were slightly younger, had a higher rate of obesity, higher TC, and lower SBP and HDL levels than those without EDS. No differences were detected in WHR, DBP, glucose, TG, or LDL (p > 0.05). In the OSA group (Table 4S), 1,058 (36.69%) men reported EDS, with a median age of 37 years (interquartile range: 32–46 years). On all measured parameters, men with EDS differed from men without EDS (p < 0.05).

Demographic data for men with and without MetS are presented in Table 2S and 5S. In the health screening examination group (Table 2S), 1,182 (36.88%) men reported MetS. Men with MetS were older, had a higher BMI, were more likely to have central obesity, and were more likely to have a worse cardio-metabolic status than men without MetS. In the OSA group (Table 5S), 1,368 (47.43%) men reported MetS. Compared to men without MetS, all measured parameters in men with EDS and MetS were altered.

*Associations between micro-arousals with EDS and Mets in both genders of OSA group*

The relationships between sleep apnea index with EDS and Mets in both genders of OSA group are shown in Table 6S. In total, micro-arousals were associated with both EDS and MetS (OR=1.015, 95%CI:1.011-1.018, P<0.001; OR=1.011, 95%CI:1.008-1.015, P<0.001) after adjusting for age, BMI, and even further adjust for smoke and drink (OR=1.014, 95%CI:1.011-1.017, P<0.001; OR=1.011, 95%CI:1.007-1.014, P<0.001). When stratified by sex, micro-arousals were only also associated with both EDS and MetS in male after adjusting for age, BMI, smoke and drink, but not in females.

*Basic characteristics of patients undergoing surgery for OSA*

The detailed demographic characteristics of subjects with and without EDS who underwent upper-airway surgery are shown in Table 7S. A total of 57 subjects with OSA (24 without EDS and 33 with EDS) were included in the analysis. The preoperative mean age was 40.27 years (39.43 years in non-EDS and 40.88 years in EDS subjects). The mean ESS score was 11.82 (5.13 in non-EDS and 16.83 in EDS subjects). The mean follow-up time was about 31.72 months (33.76 months in non-EDS and 30.24 months in EDS subjects). The sleep variables of these subjects are also presented.

Table1S Characteristics of men and women, with and without EDS in health screening examinations group

|  | Women | | | | Men | | | |
| --- | --- | --- | --- | --- | --- | --- | --- | --- |
|  | All subjects (3110) | Non-EDS (2877) | EDS (233) | p | All subjects (3202) | Non-EDS (2852) | EDS (350) | p |
| Age (yrs) | 53.00(41.00~57.00) | 53.00(41.00~57.00) | 53.00(40.00~57.00) | 0.789 | 49.00(39.00~59.00) | 49.00(39.00~60.00) | 48.00(38.75~57.00) | 0.038* |
| BMI(kg/m^2^) | 22.4(20.50~24.60) | 22.40(20.50~24.60) | 22.40(20.50~24.55) | 0.956 | 24.30(22.30~26.30) | 24.20(22.20~26.20) | 24.85(22.88~27.10) | <0.001* |
| NC (cm) | 32.00(30.00~33.00) | 32.00(30.00~33.00) | 31.00(30.00~33.00) | 0.579 | 36.00(34.00~38.00) | 36.00(34.00~38.00) | 37.00(35.00~38.00) | <0.001* |
| WC (cm) | 75.00(69.00~81.00) | 75.00(69.00~81.00) | 76.00(69.50~81.50) | 0.352 | 84.00(78.00~90.00) | 84.00(78.00~90.00) | 86.00(79.00~92.00) | 0.005* |
| HC (cm) | 90.00(86.00~95.00) | 90.00(86.00~95.00) | 91.00(86.00~95.00) | 0.446 | 94.00(90.00~98.00) | 94.00(90.00~98.00) | 95.50(91.00~100.00) | <0.001* |
| WHR | 0.82(0.78~0.87) | 0.82(0.78~0.87) | 0.83(0.78~0.88) | 0.396 | 0.89(0.85~0.93) | 0.89(0.86~0.93) | 0.90(0.85~0.93) | 0.706 |
| SBP (mmHg) | 125.00(112.00~141.00) | 125.00(112.00~142.00) | 122.00(111.00~136.00) | 0.083 | 131.00(119.00~145.00) | 131.00(119.00~146.00) | 127.50(117.75~141.00) | 0.008* |
| DBP(mmHg) | 75.00(67.00~84.00) | 76.00(67.00~84.00) | 73.00(65.50~82.00) | 0.039* | 80.00(72.00~88.00) | 80.00(72.00~88.00) | 79.00(72.00~86.25) | 0.088 |
| ESS | 4.00(1.00~6.00) | 3.00(0.00~6.00) | 13.00(12.00~14.00) | <0.001* | 4.00(2.00~8.00) | 4.00(2.00~6.00) | 13.00(11.75~15.00) | <0.001* |
| Glucose (mmol/L) | 5.16(4.88~5.51) | 5.16(4.88~5.51) | 5.16(4.86~5.47) | 0.721 | 5.24(4.93~5.67) | 5.25(4.93~5.66) | 5.24(4.97~5.71) | 0.809 |
| TC(mmol/L) | 1.06(0.76~1.58) | 1.07(0.76~1.60) | 1.01(0.71~1.42) | 0.029* | 1.43(0.97~2.11) | 1.42(0.96~2.10) | 1.52(1.05~2.20) | 0.014* |
| TG(mmol/L) | 4.91(4.31~5.58) | 4.91(4.31~5.60) | 4.90(4.30~5.47) | 0.338 | 4.76(4.18~5.33) | 4.76(4.18~5.34) | 4.81(4.22~5.31) | 0.662 |
| HDL(mmol/L) | 1.49(1.29~1.73) | 1.49(1.29~1.73) | 1.50(1.32~1.76) | 0.304 | 1.20(1.04~1.41) | 1.20(1.05~1.41) | 1.19(1.01~1.36) | 0.019* |
| LDL(mmol/L) | 3.25(2.65~3.96) | 3.26(2.65~3.98) | 3.20(2.60~3.82) | 0.207 | 3.30(2.75~3.91) | 3.29(2.75~3.91) | 3.36(2.76~3.90) | 0.697 |
| Metabolic score | 1.00(0~2.00) | 1.00(0~2.00) | 0(0~1) | 0.054 | 2.00(1.00~3.00) | 2.00(1.00~3.00) | 2.00(1.00~3.00) | 0.014* |
| Non-smoker, N(%) | 3071(98.7%) | 2840(98.7%) | 231(99.1%) | 0.573 | 1715(53.6%) | 1553(54.5%) | 162(46.3%) | 0.004* |
| Non-drinker, N(%) | 2956(95.0%) | 2738(95.2%) | 218(93.6%) | 0.277 | 1693(52.9%) | 1539(54.0%) | 154(44%) | <0.001* |

Note: *p < 0.05;

Abbreviations: EDS, excessive daytime sleepiness; BMI, body mass index; NC, neck circumference; WC, waist circumference; HC, hip circumference; TC, total cholesterol; TG, triglycerides; HDL-C, high-density lipoprotein cholesterol; LDL-C, low-density lipoprotein cholesterol; SBP, systolic blood pressure; DBP, diastolic blood pressure; ESS, Epworth Sleepiness Scale.

Table 2S Characteristics of men and women, with and without MetS in health screening examination group

|  | Women | | | | Men | | | |
| --- | --- | --- | --- | --- | --- | --- | --- | --- |
|  | All subjects (3110) | Non-MetS (2881) | MetS (229) | p | All subjects (3202) | Non-MetS (2021) | MetS (1181) | p |
| Age (yrs) | 53.00(41.00~57.00) | 52.00(41.00~57.00) | 57.00(54.00~64.50) | <0.001* | 49.00(39.00~59.00) | 48.00(37.50~59.00) | 50.00(41.00~60.00) | <0.001* |
| BMI (kg/m2) | 22.4(20.50~24.60) | 22.30(20.40~24.40) | 24.80(22.90~26.65) | <0.001* | 24.30(22.30~26.30) | 23.60(21.70~25.60) | 25.30(23.60~27.10) | <0.001* |
| NC (cm) | 32.00(30.00~33.00) | 31.00(30.00~33.00) | 33.00(32.00~35.00) | <0.001* | 36.00(34.00~38.00) | 36.00(34.00~37.00) | 37.00(35.00~39.00) | <0.001* |
| WC(cm) | 75.00(69.00~81.00) | 74.00(69.00~80.00) | 82.00(76.50~87.00) | <0.001* | 84.00(78.00~90.00) | 82.00(76.00~88.00) | 88.00(82.00~92.00) | <0.001* |
| HC(cm) | 90.00(86.00~95.00) | 90.00(86.00~94.00) | 93.00(90.00~98.00) | <0.001* | 94.00(90.00~98.00) | 93.00(89.00~97.50) | 95.00(92.00~100.00) | <0.001* |
| WHR | 0.82(0.78~0.87) | 0.82(0.78~0.86) | 0.87(0.83~0.90) | <0.001* | 0.89(0.85~0.93) | 0.88(0.84~0.92) | 0.91(0.88~0.95) | <0.001* |
| SBP (mmHg) | 125.00(112.00~141.00) | 123.00(112.00~140.00) | 144.00(128.00~159.50) | <0.001* | 131.00(119.00~145.00) | 128.00(117.00~141.00) | 135.00(122.00~151.00) | <0.001* |
| DBP(mmHg) | 75.00(67.00~84.00) | 75.00(66.00~83.00) | 83.00(76.00~90.00) | <0.001* | 80.00(72.00~88.00) | 78.00(71.00~86.00) | 83.00(76.00~92.00) | <0.001* |
| ESS | 4.00(1.00~6.00) | 4.00(1.00~6.00) | 3.00(1.00~6.00) | 0.136 | 4.00(2.00~8.00) | 4.00(2.00~7.00) | 5.00(2.00~8.00) | 0.001* |
| Glucose (mmol/L) | 5.16(4.88~5.51) | 4.14(4.87~5.45) | 6.29(5.32~7.27) | <0.001* | 5.24(4.93~5.67) | 5.16(4.88~5.48) | 5.49(5.06~6.28) | <0.001* |
| TC(mmol/L) | 1.06(0.76~1.58) | 1.01(0.73~1.44) | 2.58(2.09~3.42) | <0.001* | 1.43(0.97~2.11) | 1.11(0.82~1.43) | 2.28(1.86~3.10) | <0.001* |
| TG(mmol/L) | 4.91(4.31~5.58) | 4.89(4.29~5.56) | 5.10(4.55~5.81) | <0.001* | 4.76(4.18~5.33) | 4.65(4.10~5.23) | 4.93(4.34~5.47) | <0.001* |
| HDL(mmol/L) | 1.49(1.29~1.73) | 1.52(1.33~1.75) | 1.01(0.92~1.23) | <0.001* | 1.20(1.04~1.41) | 1.33(1.16~1.51) | 1.05(0.94~1.17) | <0.001* |
| LDL(mmol/L) | 3.25(2.65~3.96) | 3.22(2.62~3.94) | 3.57(3.08~4.21) | <0.001* | 3.30(2.75~3.91) | 3.18(2.64~3.77) | 3.51(2.95~4.09) | <0.001* |
| Metabolic score | 1.00(0~2.00) | 0(0~1.00) | 3.00(3.00~4.00) | <0.001* | 2.00(1.00~3.00) | 2.00(1.00~2.00) | 3.00(3.00~4.00) | <0.001* |
| Non-smoker, N(%) | 3071(98.7%) | 2848(98.9%) | 223(97.4%) | 0.054 | 1715(53.6%) | 1149(56.9%) | 566(47.9%) | <0.001* |
| Non-drinker, N(%) | 2956(95.0%) | 2736(95.0%) | 220(96.1%) | 0.459 | 1693(52.9%) | 1120(55.4%) | 573(48.5%) | <0.001* |

Note: *p < 0.05;

Abbreviations: MetS, metabolic syndrome; BMI, body mass index; NC, neck circumference; WC, waist circumference; HC, hip circumference; TC, total cholesterol; TG, triglycerides; HDL-C, high-density lipoprotein cholesterol; LDL-C, low-density lipoprotein cholesterol; SBP, systolic blood pressure; DBP, diastolic blood pressure; ESS, Epworth Sleepiness Scale.

Table 3S Basic characteristics of subjects in OSA group

|  | Non-OSA  (N=790) | Mild OSA  (N=617) | Moderate OSA  (N=555) | Severe OSA  (N=1616) | P |
| --- | --- | --- | --- | --- | --- |
| Age (yrs) | 36(30~46) | 39(32~49) | 40(34~51) | 39(33~47) | <0.001 |
| Male(%) | 460(58.2%) | 478(77.5%) | 464(83.6%) | 1482(91.7%) | <0.001 |
| BMI (kg/m2) | 23.44(21.48~25.39) | 24.91(23.01~27.05) | 25.71(24.0~27.73) | 27.55(25.48~29.76) | <0.001 |
| NC (cm) | 36(33~58) | 38(36~40) | 39(37~41) | 40.5(39~42) | <0.001 |
| WC (cm) | 85(79~91) | 91(85~96) | 94(88~98) | 98(93~104) | <0.001 |
| HC(cm) | 96(92~100) | 98(94~102) | 99.5(96~104) | 102(98~107) | <0.001 |
| WHR | 0.89(0.84~0.93) | 0.92(0.89~0.96) | 0.94(0.91~0.97) | 0.96(0.93~0.99) | <0.001 |
| SBP(mmHg) | 120(110~125) | 120(114~129) | 121(114~131) | 124(118~134) | <0.001 |
| DBP(mmHg) | 77(70~80) | 78(70~81) | 79(70~85) | 80(75~88) | <0.001 |
| ESS | 4(0~8) | 7(3~10) | 7(3~11) | 10(6~15) | <0.001 |
| Non-smoker, N(%) | 629(79.6%) | 402(65.2%) | 317(57.1%) | 823(51.0%) | <0.001 |
| Non-drinker, N(%) | 676(85.6%) | 498(80.7%) | 450(81.1%) | 1269(78.5%) | <0.001 |
| Glucose (mmol/L) | 4.98(4.64~5.29) | 5.1(4.8~5.4) | 5.18(4.87~5.56) | 5.33(4.98~5.79) | <0.001 |
| Insulin (μU/mL) | 7.22(4.98~10.27) | 8.44(5.77~12.95) | 9.94(6.82~14.16) | 12.6(8.57~18.06) | <0.001 |
| HOMA-IR | 1.58(1.04~2.32) | 1.89(1.25~2.95) | 2.30(1.53~3.30) | 2.99(1.99~4.60) | <0.001 |
| TC(mmol/L) | 4.29(3.74~4.87) | 4.59(4.04~5.22) | 4.69(4.15~5.28) | 4.84(4.27~5.43) | <0.001 |
| TG(mmol/L) | 1.06(0.72~1.54) | 1.31(0.91~1.93) | 1.55(1.08~2.28) | 1.75(1.24~2.57) | <0.001 |
| HDL(mmol/L) | 1.11(0.96~1.30) | 1.09(0.93~1.26) | 1.03(0.9~1.21) | 1(0.89~1.14) | <0.001 |
| LDL(mmol/L) | 2.61(2.12~3.14) | 2.91(2.41~3.4) | 2.97(2.52~3.46) | 3.08(2.6~3.61) | <0.001 |
| AHI | 1.4(0.5~2.9) | 9(6.8~12.0) | 21.6(17.9~25.5) | 58.3(44.4~70.6) | <0.001 |
| LSpO_2_ | 94(91~96) | 88(84~91) | 83(78~88) | 72(63~79) | <0.001 |
| ODI | 1.5(0.6~3.3) | 9.1(6.3~12.5) | 21.7(16.9~27.4) | 59.4(43.8~73.5) | <0.001 |
| MAI | 12.2(7.8~19.7) | 17.1(10.3~24.8) | 20.7(11.8~30.5) | 36.1(18.7~55.5) | <0.001 |
| Metabolic score | 1(0~2) | 2(1~3) | 2(2~3) | 3(2~3) | <0.001 |
| EDS, N(%) | 124(15.7%) | 153(24.8%) | 153(27.6%) | 775(48.0%) | <0.001 |
| MetS, N(%) | 119(15.1%) | 164(26.6%) | 250(45.0%) | 928(57.4%) | <0.001 |

Table 4S Characteristics of men and women, with and without EDS in OSA group

|  | Women | | | | Men | | | |
| --- | --- | --- | --- | --- | --- | --- | --- | --- |
|  | All subjects(694) | Non-EDS(547) | EDS(147) | p | All subjects(2884) | Non-EDS(1826) | EDS(1058) | p |
| Age (yrs) | 45.00(35.00~55.00) | 44.00(35.00~54.00) | 50.00(37.00~58.00) | 0.002* | 37.00(32.00~46.00) | 37.00(31.00~45.00) | 39.00(33.00~46.00) | 0.001* |
| BMI (kg/m2) | 23.90(21.67~26.56) | 23.63(21.48~26.03) | 24.84(22.38~28.89) | <0.001* | 26.26(24.16~28.60) | 25.83(23.66~28.01) | 27.22(25.17~29.41) | <0.001* |
| NC (cm) | 34.00(32.00~36.00) | 34.00(32.00~36.00) | 35.00(33.00~38.00) | <0.001* | 40.00(38.00~42.00) | 39.06(38.00~41.00) | 40.00(38.50~42.00) | <0.001* |
| WC (cm) | 85.50(78.00~93.00) | 85.00(77.00~91.00) | 90.00(82.50~100.00) | <0.001* | 95.00(90.00~101.00) | 94.00(88.00~100.00) | 97.00(91.00~104.00) | <0.001* |
| HC(cm) | 96.00(92.00~101.00) | 96.00(91.00~100.00) | 99.00(92.00~105.00) | <0.001* | 100.00(96.00~105.00) | 100.00(96.00~104.00) | 102.00(97.00~106.00) | <0.001* |
| WHR | 0.89(0.84~0.93) | 0.88(0.84~0.92) | 0.92(0.85~0.96) | <0.001* | 0.95(0.91~0.98) | 0.94(0.90~0.97) | 0.96(0.92~0.99) | <0.001* |
| SBP(mmHg) | 120.00(110.00~127.00) | 120.00(112.00~126.00) | 120.00(110.00~128.00) | 0.622 | 121.00(116.00~131.00) | 121.00(115.00~130.00) | 123.00(116.00~133.00) | 0.004* |
| DBP(mmHg) | 77.00(69.00~81.00) | 77.00(70.00~80.00) | 76.00(67.00~83.00) | 0.875 | 37.00(32.00~46.00) | 80.00(72.00~84.00) | 80.00(74.00~87.00) | <0.001* |
| ESS | 5.00(1.00~9.00) | 3.00(0~7.00) | 13.00(12.00~16.00) | <0.001* | 8.00(4.00~13.00) | 5.00(2.00~8.00) | 14.00(12.00~17.00) | <0.001* |
| Glucose (mmol/L) | 5.01(4.69~5.42) | 7.82(5.15~11.92) | 5.10(4.73~5.60) | 0.009* | 5.20(4.88~5.60) | 5.17(4.86~5.52) | 5.28(4.95~5.76) | <0.001* |
| Insulin (μU/mL) | 8.00(5.37~12.14) | 1.73(1.12~11.92) | 8.48(6.11~12.94) | 0.069 | 10.51(6.91~15.57) | 9.83(6.51~14.56) | 11.59(7.91~11.12) | <0.001* |
| HOMA-IR | 1.77(1.16~2.82) | 1.73(1.12~2.77) | 1.95(1.32~3.08) | 0.023* | 2.44(1.58~3.76) | 2.25(1.47~3.46) | 2.81(1.81~4.34) | <0.001* |
| TC(mmol/L) | 4.56(3.87~5.22) | 4.53(3.84~5.15) | 4.78(4.19~5.50) | 0.002* | 4.67(4.11~5.30) | 4.59(4.04~5.23) | 4.79(4.24~5.41) | <0.001* |
| TG(mmol/L) | 1.08(0.74~1.53) | 1.06(0.72~1.52) | 1.16(0.81~1.63) | 0.047* | 1.59(1.11~2.33) | 1.52(1.03~2.18) | 1.76(1.24~2.62) | <0.001* |
| HDL(mmol/L) | 1.19(1.04~1.41) | 1.18(1.03~1.40) | 1.25(1.07~1.46) | 0.083 | 1.01(0.89~1.15) | 1.02(0.90~1.16) | 1.00(0.88~1.13) | 0.006** |
| LDL(mmol/L) | 2.78(2.23~3.35) | 2.74(2.19~3.30) | 3.03(2.38~3.54) | 0.002* | 2.98(2.48~3.50) | 2.93(2.46~3.41) | 3.07(2.55~3.63) | <0.001* |
| AHI | 5.85(1.00~22.88) | 4.00(0.80~17.20) | 20.70(4.80~45.40) | <0.001* | 31.50(9.70~59.10) | 21.65(6.58~48.83) | 50.95(21.98~68.20) | <0.001* |
| LSpO_2_ | 90.00(81.00~94.00) | 91.00(84.00~95.00) | 82.00(73.00~91.00) | <0.001* | 81.00(70.00~89.00) | 84.00(75.00~90.00) | 75.00(64.00~84.00) | <0.001* |
| ODI | 5.75(1.20~25.60) | 4.30(1.00~19.30) | 20.30(4.00~49.50) | <0.001* | 30.75(9.10~60.20) | 21.00(6.38~48.85) | 50.00(20.38~69.90) | <0.001* |
| MAI | 13.20(7.70~23.55) | 12.70(7.60~21.90) | 16.50(8.70~31.60) | 0.015* | 23.50(12.90~42.00) | 21.05(12.00~36.40) | 29.90(15.90~53.57) | <0.001* |
| Metabolic score | 1.00(0.00~2.00) | 1.00(0.00~2.00) | 1.00(0~2.00) | 0.063 | 2.00(2.00~3.00) | 2.00(1.00~3.00) | 3.00(2.00~3.00) | <0.001* |
| Non-smoker, N(%) | 670(96.5%) | 530(96.9%) | 140(95.2%) | 0.330 | 1501(52.0%) | 996(54.5%) | 505(47.7%) | <0.001* |
| Non-drinker, N(%) | 650(93.7%) | 519(94.9%) | 131(89.1%) | 0.011* | 2243(77.8%) | 1440(78.9%) | 803(75.9%) | 0.065 |

Note: *p < 0.05;

Abbreviations: EDS, excessive daytime sleepiness; OSA, obstructive sleep apnea; BMI, body mass index; NC, neck circumference; WC, waist circumference; HC, hip circumference; HOMA-IR, homeostasis model of assessment for insulin resistance index; TC, total cholesterol; TG, triglycerides; HDL-C, high-density lipoprotein cholesterol; LDL-C, low-density lipoprotein cholesterol; SBP, systolic blood pressure; DBP, diastolic blood pressure; LSpO_2_, lowest pulse oxygen saturation; ODI, oxygen desaturation index; MAI, micro-arousal index; AHI, apnea-hypopnea index; ESS, Epworth Sleepiness Scale.

Table 5S Characteristics of men and women, with and without MetS in OSA group

|  | Women | | | | Men | | | |
| --- | --- | --- | --- | --- | --- | --- | --- | --- |
|  | All subjects(694) | Non-MetS(601) | MetS(93) | p | All subjects(2884) | Non-MetS(1516) | MetS(1368) | p |
| Age (yrs) | 45.00(35.00~55.00) | 44.00(34.00~54.00) | 49.00(43.00~57.00) | 0.01* | 37.00(32.00~46.00) | 37.00(31.00~46.00) | 38.00(33.00~45.00) | 0.002* |
| BMI(kg/m2) | 23.90(21.67~26.56) | 23.42(21.24~25.64) | 27.47(25.71~30.21) | <0.001* | 26.26(24.16~28.60) | 24.75(22.89~27.10) | 27.68(25.95~29.76) | <0.001* |
| NC (cm) | 34.00(32.00~36.00) | 34.00(32.00~36.00) | 36.00(35.00,38.00) | <0.001* | 40.00(38.00~42.00) | 39.00(37.00~40.00) | 41.00(39.00~43.00) | <0.001* |
| WC(cm) | 85.50(78.00~93.00) | 84.00(77.00~90.00) | 94.50(89.50~100.50) | <0.001* | 95.00(90.00~101.00) | 90.00(86.00~96.00) | 99.00(94.00~105.00) | <0.001* |
| HC(cm) | 96.00(92.00~101.00) | 96.00(91.00~100.00) | 100.00(95.85~106.00) | <0.001* | 100.00(96.00~105.00) | 98.00(95.00~102.00) | 103.00(99.00~107.00) | <0.001* |
| WHR | 0.89(0.84~0.93) | 0.88(0.83~0.92) | 0.95(0.91~0.97) | <0.001* | 0.95(0.91~0.98) | 0.93(0.89~0.96) | 0.96(0.94~1.00) | <0.001* |
| SBP(mmHg) | 120.0(110.0~127.0) | 120.0(110.0~125.0) | 129.0(116.0~137.0) | <0.001* | 121.0(116.0~131.0) | 120.0(113.0~126.0) | 127.0(120.0~137.0) | <0.001* |
| DBP(mmHg) | 77.00(69.00~81.00) | 76.00(69.00~80.00) | 80.00(74.00~89.00) | <0.001* | 80.00(73.00~85.00) | 78.00(70.00~80.00) | 81.00(76.00~90.00) | <0.001* |
| ESS | 5.00 (1.00~9.00) | 5.00(1.00~9.00) | 7.00(2.00~11.50) | 0.020* | 8.00(4.00~13.00) | 7.00(3.00~11.00) | 10.00(5.00~14.00) | <0.001* |
| Glucose (mmol/L) | 5.01(4.69~5.42) | 4.97(4.66~5.33) | 5.63(5.01~6.53) | <0.001* | 5.20(4.88~5.60) | 5.08(4.80~5.37) | 5.39(5.03~6.02) | <0.001** |
| Insulin (μU/mL) | 8.00(5.37~12.14) | 7.57(5.09~11.09) | 12.40(8.61~20.01) | <0.001* | 10.51(6.91~15.57) | 8.32(5.68~12.13) | 13.29(9.50~18.77) | <0.001* |
| HOMA-IR | 1.77(1.16~2.82) | 1.65(1.11~2.46) | 3.39(2.02~4.93) | <0.001* | 2.44(1.58~3.76) | 1.86(1.24~2.79) | 3.20(2.20~4.77) | <0.001* |
| TC(mmol/L) | 4.56(3.87~5.22) | 4.53(3.84~5.19) | 4.79(4.24~5.32) | 0.017* | 4.67(4.11~5.30) | 4.46(3.96~5.07) | 4.90(4.35~5.51) | <0.001* |
| TG(mmol/L) | 1.08(0.74~1.53) | 1.01(0.70~1.35) | 2.05(1.61~2.86) | <0.001* | 1.59(1.11~2.33) | 1.20(0.88~1.52) | 2.26(1.80~3.03) | <0.001* |
| HDL(mmol/L) | 1.19(1.04~1.41) | 1.23(1.08~1.45) | 0.97(0.88~1.09) | <0.001* | 1.01(0.89~1.15) | 1.08(0.94~1.25) | 0.96(0.85~1.07) | <0.001* |
| LDL(mmol/L) | 2.78(2.23~3.35) | 2.77(2.19~3.35) | 2.81(2.37~3.38) | 0.260 | 2.98(2.48~3.50) | 2.92(2.44~3.40) | 3.04(2.54~3.59) | <0.001* |
| AHI | 5.85(1.00~22.88) | 4.30(0.90~19.90) | 21.50(6.20~44.60) | <0.001* | 31.50(9.70~59.10) | 18.35(5.20~46.48) | 46.95(20.53~66.60) | <0.001* |
| LSpO_2_ | 90.0(81.0~94.0) | 91.00(82.00~94.00) | 84.00(71.00~90.50) | <0.001* | 81.00(70.00~89.00) | 85.00(75.00~91.00) | 76.00(65.00~85.00) | <0.001* |
| ODI | 5.75(1.20~25.60) | 4.50(1.00~21.20) | 23.30(7.50~60.80) | <0.001* | 30.75(9.10~60.20) | 16.95(4.70~45.10) | 47.82(19.98~68.70) | <0.001* |
| MAI | 13.20(7.70~23.55) | 13.00(7.70~23.20) | 15.70(7.65~25.50) | 0.268 | 23.50(12.90~42.00) | 20.20(11.90~35.48) | 29.05(14.93~49.90) | <0.001* |
| Metabolic score | 1.00(0.00~2.00) | 1.00(0.00~1.50) | 3.00(3.00~3.00) | <0.001* | 2.00(2.00~3.00) | 2.00(1.00~2.00) | 3.00(3.00~4.00) | <0.001* |
| Non-smoker, N(%) | 670(96.5%) | 581(96.7%) | 89(96.7%) | 0.633 | 1501(52.0%) | 872(57.5%) | 629(50.0%) | <0.001* |
| Non-drinker, N(%) | 650(93.7%) | 562(93.5%) | 88(94.6%) | 0.682 | 2243(77.8%) | 1178(77.7%) | 1065(77.9%) | 0.925 |

Note: *p < 0.05;

Abbreviations: MetS, metabolic syndrome; OSA, obstructive sleep apnea; BMI, body mass index; NC, neck circumference; WC, waist circumference; HC, hip circumference; HOMA-IR, homeostasis model of assessment for insulin resistance index; TC, total cholesterol; TG, triglycerides; HDL-C, high-density lipoprotein cholesterol; LDL-C, low-density lipoprotein cholesterol; SBP, systolic blood pressure; DBP, diastolic blood pressure; LSpO_2_, lowest pulse oxygen saturation; ODI, oxygen desaturation index; MAI, micro-arousal index; AHI, apnea-hypopnea index; ESS, Epworth Sleepiness Scale.

Table 6S The relationships between micro-arousals with EDS and Mets in both genders of OSA group.

|  | Male | | Female | | Total | |
| --- | --- | --- | --- | --- | --- | --- |
|  | OR(95%CI) | P | OR(95%CI) | P | OR(95%CI) | P |
| EDS* | 1.104 (1.010-1.018) | <0.001 | 1.009 (1.000-1.019) | 0.048 | 1.015 (1.011-1.018) | <0.001 |
| Mets* | 1.010 (1.006-1.014) | <0.001 | 0.993 (0.981-1.006) | 0.297 | 1.011 (1.008-1.015) | <0.001 |
| EDS# | 1.014 (1.010-1.017) | <0.001 | 1.008 (0.999-1.018) | 0.091 | 1.014 (1.011-1.017) | <0.001 |
| Mets# | 1.010 (1.006-1.014) | <0.001 | 0.994 (0.981-1.007) | 0.338 | 1.011 (1.007-1.014) | <0.001 |

* adjust for age, BMI

#djust for age, BMI, smoke and drink

Table 7S Baseline characteristics of patients with OSA undergoing upper-airway surgery

|  | All subjects (n=57) | OSA without EDS (n=24) | OSA with EDS (n=33) | p value |
| --- | --- | --- | --- | --- |
| Age (years) | 40.27(8.02) | 39.43(9.57) | 40.88(6.80) | 0.42 |
| WC (cm) | 98.88(6.43) | 99.09(6.37) | 98.73(6.57) | 0.89 |
| HC(cm) | 102.17(5.86) | 102.17(5.13) | 102.18(6.41) | 0.97 |
| Follow-up duration (months) | 31.72(28.25) | 33.76(22.35) | 30.24(32.09) | 0.14 |
| ESS | 11.82(6.14) | 5.83(3.24) | 16.13(3.53) | <0.01* |
| AHI | 51.53(21.36) | 46.50(19.85) | 55.15(21.96) | 0.08 |
| LSpO_2_ | 72.98% (11.43%) | 76.93%(11.90%) | 70.15% (10.36%) | <0.01* |
| ODI | 51.97(21.97) | 43.70(18.88) | 57.91(22.38) | 0.01* |
| MAI | 40.45(25.31) | 31.30(23.45) | 47.03(24.88) | 0.02* |
| Non-smoker, N(%) | 28(49.1%) | 20(50%) | 8(47.1%) | 0.839 |
| Non-drinker, N(%) | 19(33.3%) | 12(30%) | 7(41.2%) | 0.413 |

Note: *p < 0.05;

Abbreviations: OSA, obstructive sleep apnea; EDS, excessive daytime sleepiness; WC, waist circumference; HC, hip circumference; LSpO_2_, lowest pulse oxygen saturation; ODI, oxygen desaturation index; MAI, micro-arousal index; AHI, apnea-hypopnea index; ESS, Epworth Sleepiness Scale.

**Reference**

1. Consultation WHOE: **Appropriate body-mass index for Asian populations and its implications for policy and intervention strategies.** *Lancet* 2004, **363:**157-163.
